# Ultrasound-enhanced hydrogen peroxide generation of microwave plasma discharges in conductive aqueous solutions

**DOI:** 10.1016/j.ultsonch.2026.108065

**Published:** 2026-09-20

**Authors:** B. Honnorat, V. Brüser, J.F. Kolb

**Affiliations:** Leibniz Institute for Plasma Science and Technology (INP), Felix-Hausdorff-Str. 2, D-17489 Greifswald, Germany

**Keywords:** Microwave discharge, Ultrasound, Underwater plasma, Reactive oxygen species

## Abstract

This study investigated the enhancement of hydrogen peroxide (H_2_O_2_) generation through the interaction between an underwater pulsed microwave plasma discharge and high-intensity ultrasound in sodium chloride (NaCl) solutions. The objective was to enhance the H_2_O_2_ yield of the plasma treatment by simultaneously applying sonication, a technique known to influence chemical transport and reactivity.

Experimental parameters, including ultrasound amplitude, NaCl concentration, and the distance between the sonotrode and the microwave source (coupler), were systematically varied to assess their impact. The results indicate that ultrasound significantly enhances H_2_O_2_ yield compared with plasma treatment alone. The maximum production was achieved at the highest ultrasound amplitude, with a sonotrode–coupler distance of 5 mm and  plasma operation with 10 Hz microwave bursts (1950 W, duty ratio 50 %). Under these conditions, the H_2_O_2_ production rate reached $0.20\;g.h^{-1}$, with a yield of $0.18\;g.{\mathrm{kWh}}^{-1}$, corresponding to increases of +47% and +26%, respectively, compared with similar conditions without ultrasound. This resulted in an H_2_O_2_ concentration of approximately 1.5mM in 1.0L after 15 min of treatment, whereas only 0.9mM was obtained without ultrasound.

Potential mechanisms for the observed yield enhancement include improved transport of reactive species and ultrasound-induced motion of the plasma–liquid interface. The effects of microwave burst frequency and duty ratio on H_2_O_2_ production were also examined. Overall, the results suggest that coupling plasma with ultrasound is a promising approach for enhancing reactive species generation in plasma–liquid systems.

## 1 Introduction

Plasma-liquid interactions generate a broad range of reactive oxygen and nitrogen species (RONS) in aqueous media [1]. In particular, the production of O_3_, OH radicals, NO, singlet oxygen, peroxynitrite, and hydrogen peroxide (H_2_O_2_) have been detected or quantified in the liquid phase [1]. Among these species, H_2_O_2_ is an important long-lived product of plasma–liquid chemistry, and its formation has been systematically reviewed for different plasma–liquid reactor configurations [2].

At industrial scale, H_2_O_2_ is produced predominantly through the anthraquinone oxidation process, which accounts for approximately 95 % of worldwide production [3]. Alternative approaches, particularly electrochemical H_2_O_2_ synthesis through the two-electron oxygen reduction reaction and plasma technologies, have attracted attention because they can enable decentralized and on-demand production directly from water and oxygen [3], [4]. The energy yield varies considerably between these technologies. Recent process benchmarking reported energy consumptions of approximately 1.3–2.1 kWh per kg of H_2_O_2_ for the industrial anthraquinone process when the production of H_2_ by steam methane reforming is included, whereas efficient electrochemical systems typically require approximately 3.1–10 kWh.kg⁻^1^ [3]. Plasma-based H_2_O_2_ production generally exhibits substantially higher energy consumption, with reported values spanning approximately 12.8–1429 kWh.kg⁻^1^ depending on the plasma configuration [3]. These values clearly indicate that plasma processes are currently not competitive with established technologies when H_2_O_2_ production efficiency alone is considered.

Hydrogen peroxide formation in plasma–liquid systems can involve several reaction pathways. Plasma-induced dissociation of water generates reactive intermediates, particularly OH radicals, whose recombination can lead to H_2_O_2_ formation. However, the phase in which H_2_O_2_ is predominantly produced depends on the discharge configuration. In some plasma–liquid systems, H_2_O_2_ may be formed in the plasma or gas phase and subsequently transferred across the plasma–liquid interface into the liquid. Such a pathway was proposed by Winter et al. [5], who investigated the transfer of plasma-generated H_2_O_2_ from the gas phase into the liquid phase. Alternatively, H_2_O_2_ may be generated directly in the liquid through secondary reactions involving plasma-produced species, including OH radicals and solvated electrons, as discussed by Locke and Shih [2]. The relative contributions of gas-phase formation followed by interfacial transport and direct liquid-phase formation remain strongly dependent on the plasma configuration and are not yet fully established, particularly for underwater microwave discharges.

Despite their comparatively low H_2_O_2_ energy efficiency, plasma–liquid systems remain of interest because H_2_O_2_ is generally not the sole product of the treatment. Plasma can generate several short- and long-lived RONS directly at the point of treatment, which is relevant to applications such as water treatment and advanced oxidation processes [1], [2]. In this context, H_2_O_2_ can also serve as a convenient indicator of plasma-induced reactive oxygen chemistry rather than necessarily being the targeted final product.

Microwave plasma discharges are attractive for RONS production because they can deliver power in the kW range at relatively low cost and with good scalability. The high plasma density achieved under microwave excitation is also expected to promote RONS generation. We chose to generate the microwave plasma directly from the liquid because this approach couples the plasma efficiently into the liquid phase and allows higher power input than many alternative plasma systems [2].

This approach also eliminates the need for gas injection to sustain the plasma discharge. Indeed, gas injection can be costly, particularly when rare gases are used, and may also alter the chemistry of the medium. For example, it can shift solubility equilibria of O_2_ and N_2_ in the liquid, thereby influencing reaction pathways and the distribution of chemical species. When the plasma is formed from the vaporized components of the liquid itself, improved contact between the plasma and the liquid phase can also be expected, which may enhance reactive species production and increase overall energy yield.

An underwater microwave plasma discharge is initiated when the incident microwave power is sufficiently high to vaporize and ionize the liquid. These discharges have been studied for various applications, including nanoparticle synthesis [6], decomposition of clathrate hydrates [7], 1,4-dioxane [8], and degradation of persistent organic pollutants such as perfluorooctanoic acid [9].

Efforts to optimize the performance of underwater microwave discharge operation for different applications have primarily focused on electrode shape [10], [11]. Pulsed rather than continuous microwave excitation has been found preferable as it extends the lifespan of components exposed to plasma discharges [12]. Additionally, Ishijima et al. emphasized the importance of controlling and stabilizing gas bubbles formed at the interface where the microwave field interacts with the liquid or the plasma that is formed as a result [13]. Some authors have also concluded that operating under reduced pressure offers significant advantages, e.g. for phenol decomposition during plasma–liquid interactions [14]. However, the operation of an underwater microwave discharge requires adequate liquid conductivity to ensure strong initial microwave absorption and to sustain plasma formation. Because conductivity can also influence reaction chemistry, the plasma was generated in NaCl solutions with different conductivities.

Reactive species can also be generated by ultrasonic sonication of water, provided the intensity is sufficient to induce cavitation [15]. The formation of these species by single cavitating bubbles [16] or clusters of bubbles [17] has been both experimentally measured and theoretically described. The sonolysis of water, known to produce H_2_O_2_ [18], has consequently been investigated for applications in wastewater treatment [19] and for accelerating the kinetics of various chemical reactions [20] such as pinacol coupling.

Consequently, it is conceivable that combining sonication with underwater microwave discharges could enhance the production of reactive species, potentially promoting synergistic interactions between the production mechanisms associated with the two techniques. The term “synergy” refers to a situation where the effect of the combined treatment is greater than the arithmetic sum of the individual treatments. Accordingly, combinations of ultrasound and plasma have already been investigated for several applications. This approach has been used to produce amorphous carbon [21]. Enhanced production of H_2_ and other fuel gases such as CH_4_, C_3_H_6_, C_3_H_4_ and N_2_H_4_ from oil has also been reported [22]. Syahrial et al. showed that the coupling of ultrasound with an underwater radiofrequency discharge increased the decomposition rate of a glucose solution [23]. Takahashi et al. demonstrated synergistic enhancement of both the decomposition rate and energy yield for methylene blue degradation [24]. Synergistic effects have also been reported for the inactivation of microorganisms when ultrasound was combined with plasma [25].

Based on the established ability of ultrasound to enhance chemical reactivity and mass transport, we investigated whether high-intensity 20 kHz ultrasound can synergistically enhance H_2_O_2_ generation during underwater microwave plasma treatment of conductive aqueous solutions H_2_O_2_ was used as an indicator of the reactive oxygen chemistry occurring during plasma–liquid interaction. However, the objective of the present study is not to demonstrate a competitive process for H_2_O_2_ production, but to investigate whether ultrasound can enhance reactive-species production. NaCl solutions were selected to provide the electrical conductivity required for stable microwave plasma formation, while also allowing the influence of liquid conductivity on H_2_O_2_ production to be assessed. The sonotrode was positioned near the microwave coupler to expose the discharge region and plasma–liquid interface to ultrasound. The effects of ultrasound operation were evaluated by varying the sonotrode–coupler distance, ultrasound amplitude, NaCl concentration, and microwave burst parameters. In addition to H_2_O_2_ quantification, discharge morphology, reflected microwave energy, and time-integrated light emission were examined to identify how ultrasound modifies the plasma–liquid interaction.

## 2 Materials and methods

The complete dataset supporting this study, together with the supplementary materials, is available at https://doi.org/10.17632/h9s884wsnc.2. A plasma was formed in the liquid by the breakdown of the electric field that was transmitted into the aqueous solution by a microwave coupler. The microwave source was a magnetron (GMP-20-KED, SAIREM), delivering 1950 W at 2.45 GHz through WR340 waveguides (Fig. 1a). The microwave circuit included a circulator to protect the magnetron from reflected power, a directional coupler for measuring forward and reflected power, and two tuning elements: a sliding short-circuit and a manual three-stub tuner. The directional coupler included two 60 dB attenuators to select the forward and backward power, connected to high-frequency diodes that converted the microwave signals into voltages proportional to the forward and backward power. The signals of the diodes were recorded on an oscilloscope (WaveSurfer 64MXs-B, 600 MHz, LeCroy) and subsequently processed with Jupyter [26]. The diodes were calibrated with a vector network analyzer (ZVRE, Rohde & Schwarz, 1043.009).

**Fig. 1:**
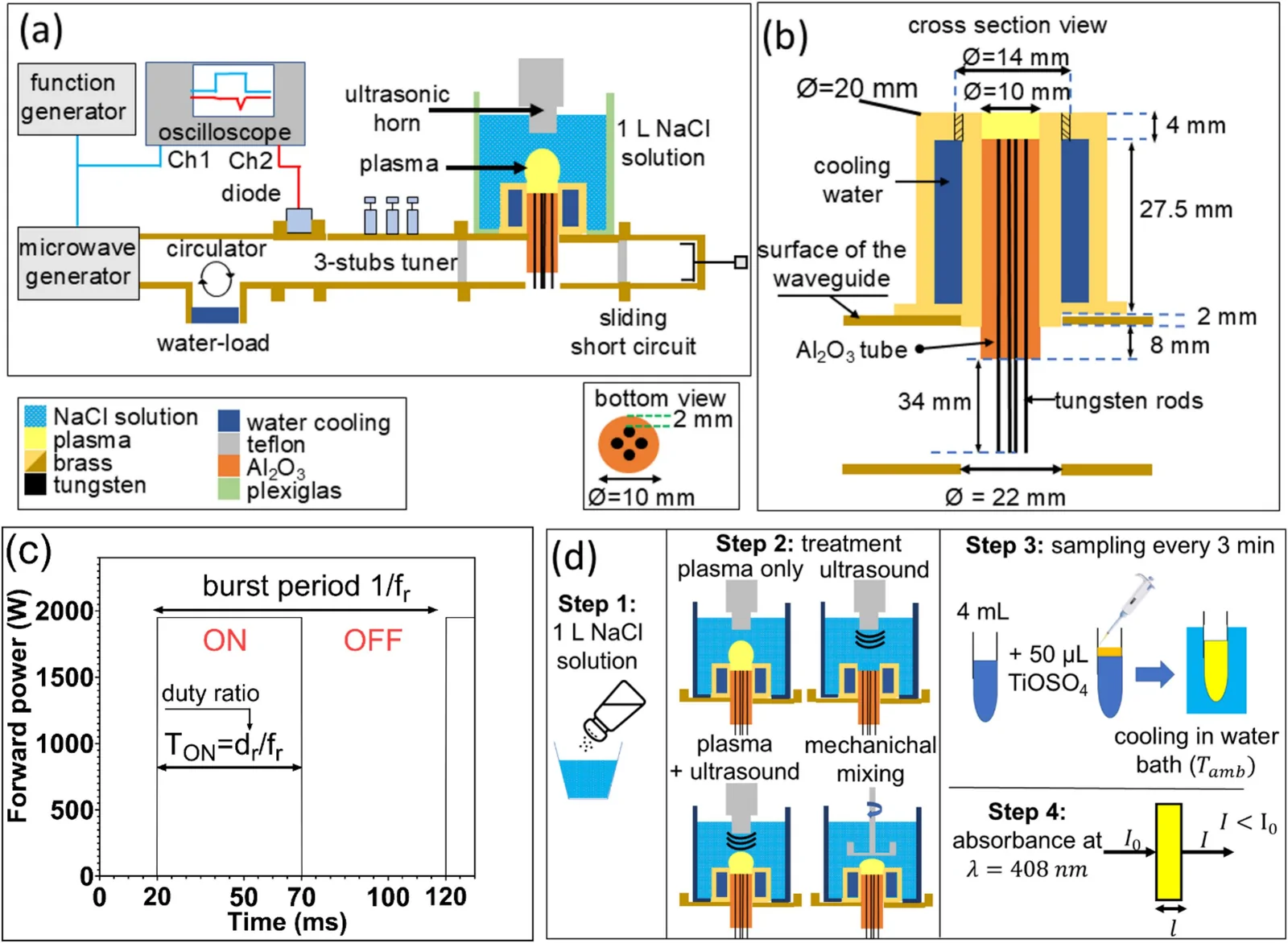
Overview of the overall microwave setup and experimental procedure: (a) microwave circuit used for plasma generation; (b) cross-sectional view of the water-cooled coupler that transmits microwaves from the inner part of the waveguide to the liquid. (c) Forward power of the microwave bursts, shown for a burst repetition frequency $f_{r}=10\;Hz$, a duty ratio $d_{r}=50\;\%,$ and an instantaneous microwave power $P_{i}=1950\;W$; (d) Steps of the experimental protocol to measure H_2_O_2_ production for the different treatment modalities (plasma only, ultrasound only, plasma combined with ultrasound, plasma combined with mechanical stirring).

The reflected energy per burst was determined through numerical integration. The code used for data processing is provided in the supplementary material. The mean energy and its standard error of the mean were calculated from 200 measurements (Table 1).

Tuning components were used to minimize the reflected power. A function generator (FY3200S, Feeltech) controlled the microwave burst timing and the microwave power. The microwave bursts are defined by three parameters: the instantaneous microwave power $P_{i}$, the burst repetition frequency $f_{r}$, and the duty ratio $d_{r}$ (Fig. 1c). Most of the experiments were conducted with the following parameters: $f_{r}=10\;\mathrm{Hz}$, $d_{r}=50\;\%$, $P_{i}=1950\;W$. Under these conditions, each microwave burst lasted for $T_{\mathrm{ON}}=d_{r}/f_{r}=50\;\mathrm{ms}$ and delivered 98 J of microwave energy. Two 1-cm thick PTFE windows protected the magnetron from an accidental inner-waveguide discharge and liquid leakage.

The coupler was designed to transfer power from the inner waveguide into the liquid (Fig. 1b). The outer conductor of the coupler was made of brass. An Al_2_O_3_ tube (99.7 % purity, ALM102FOUR, Almath Crucibles) of 10  mm in diameter and a length of 37.5  mm was inserted into the brass component, leaving a 4-mm gap above the ceramic tube. Four parallel 2-mm boreholes along the Al_2_O_3_ tube each accommodated a 71.5-mm tungsten rod (Fig. 1b). The metal and ceramic components were bonded using Resbond® 3901, a ceramic adhesive, mixed with 4 wt% of hardener 3901A (Cotronics). The entire assembly was heated for 4 h at 92 °C, followed by 1 h at 400 °C, to ensure moisture resistance of the adhesive.

The microwave propagated through the ceramic, which is effectively transparent at the operating frequency, and the plasma formed at the ceramic surface. A few milliseconds after the onset of the microwave burst and, correspondingly, the ignition of the discharge, the plasma expanded into the liquid. The expansion of the plasma and bubbles was recorded with a slow-motion camera (Chronos 1.4, Kron Technologies Inc.) and a Computar 12.5–75 mm, f/1.2 zoom lens (Kron Technologies Inc.). The frequency of acquisition and the exposure time were 15941 Hz and 58 µs, respectively. Images were processed using ImageJ [27]. The supplementary material also includes the ImageJ code for image processing. For each image, the pixel intensities of the recorded discharges were summed, and it was verified that the maximum intensity remained below the saturation limit of the sensor. The emitted light was then integrated over time. The values reported in Table 1 are averages of 20 individual microwave bursts.

In addition, discharges were observed with a standard digital camera (SM-G988B, Samsung) as shown in Fig. 2a-c for an exposure time of 10 ms. A video of the discharge, recorded for the same exposure time, is also available in the supplementary material.

Ultrasound was generated using a Q700 sonicator (Qsonica) driving a CL-334 sonotrode at 20 kHz. The portion of the sonotrode submerged in the liquid was cylindrical, with a diameter of one inch. The sonicator also controlled the vibration amplitude at the tip of the sonotrode and monitored the ultrasonic power transmitted to the solution. According to the specifications of the sonotrode, an amplitude setting of 100 % corresponds to a maximum displacement of 35 µm, with the amplitude adjustable from 1 % to 100 %. At 100 % amplitude, the transmitted ultrasonic power was approximately 140 W, whereas at 50 % amplitude, it was 49.3 W. The position of the sonotrode relative to the microwave coupler did not affect the delivered ultrasonic power.

NaCl solutions were prepared using ACS reagent-grade sodium chloride (≥99.0 % purity, S9888, Sigma-Aldrich) dissolved in deionized water.

The quantification of H_2_O_2_ was performed using the TiOSO_4_ method [28]. H_2_O_2_ reacts with TiOSO_4_ to form an absorbing complex, which is indicated by a yellow color. The assay was performed by mixing 50 µL of the TiOSO_4_ solution (89532–1 L, Sigma-Aldrich) with 4 mL of the sample. The amount of H_2_O_2_ was quantified from absorbance at λ=408nm in a 1-cm optical path cuvette (spectrophotometer Evolution 300, UV–vis, Thermo Scientific). The method was calibrated using H_2_O_2_ standards prepared in NaCl solution. All measurements fell within the linear range of this TiOSO_4_ calibration curve. The H_2_O_2_ concentration was monitored every 3 min during the treatment. For each 4-mL sample, both the microwave discharge and ultrasound generation were paused, and the reactor was stirred prior to sampling.

Statistical analyses were performed using GraphPad Prism for Windows, 64-bit version 11.0.2 (92). GraphPad Prism was also used to perform linear regression of the initial H_2_O_2_ production rate. Statistical significance was indicated as follows: n.s., not significant; ∗ p < 0.05; ∗∗ p < 0.01; and ∗∗∗ p < 0.001. Pairwise statistical comparisons of the initial H_2_O_2_ production rates were performed using an unpaired *t*-test. Statistical comparisons of the initial H_2_O_2_ production rates obtained at different ultrasound amplitudes were performed using an ordinary one-way ANOVA followed by Fisher’s uncorrected least significant difference (LSD) test.

No noticeable electrode erosion was observed under the operating conditions used in this study, including after more than 8 h of cumulative treatment under similar or harsher conditions. Nevertheless, dissolved metals may be released from plasma-facing components. To assess any effect of dissolved metals on H_2_O_2_ quantification, the H_2_O_2_ concentration was measured immediately after plasma–ultrasound treatment and again 15 min later; no decrease was observed over this timescale. This suggests that the release of metal ions, such as copper ions, was limited under the present conditions.

After each sampling, 4 mL of NaCl stock solution was added to maintain a constant treatment volume of 1  L. The treatment itself did not significantly change the volume; the volume loss was estimated to be less than 10  mL. Depending on experimental conditions, NaCl concentrations were in the range from 10-25g/L. The liquid temperature increased progressively during combined plasma–ultrasound treatment and reached ∼ 40 °C after 15 min of treatment.

The conductivity, used as an indicator of the ionic content of the solution, was normalized to 25 °C to account for its temperature dependence. The temperature-corrected conductivity (2 % per °C) did not change significantly during the treatment, indicating that the ionic content of the solution remained approximately constant.

Using MQuant^TM^ test strips for nitrate and nitrite analysis (reference 1.10020.0001, VWR^TM^), nitrate and nitrite concentrations produced during the combined plasma–ultrasound treatment were below the limits of detection of 10 mg/L and 2.0 mg/L, respectively. These measurements were performed after 15 min of treatment under the following conditions: ($f_{r}=10\;Hz$, $d_{r}=50\;\%$, $P_{i}=1950\;W$), 100 % ultrasound amplitude, and a sonotrode–coupler distance of 5 mm. Under similar conditions, the pH (PHT-200, Voltcraft) showed no significant change within a precision of 0.05. These observations are consistent with previous reports [29], [30].

## 3 Results

### 3.1 Discharge morphology

The components of the experimental setup are shown in Fig. 2a, together with a time-integrated image of a discharge in NaCl solution. The sonotrode was removed to illustrate the expansion of the microwave discharge in its absence.

**Fig. 2:**
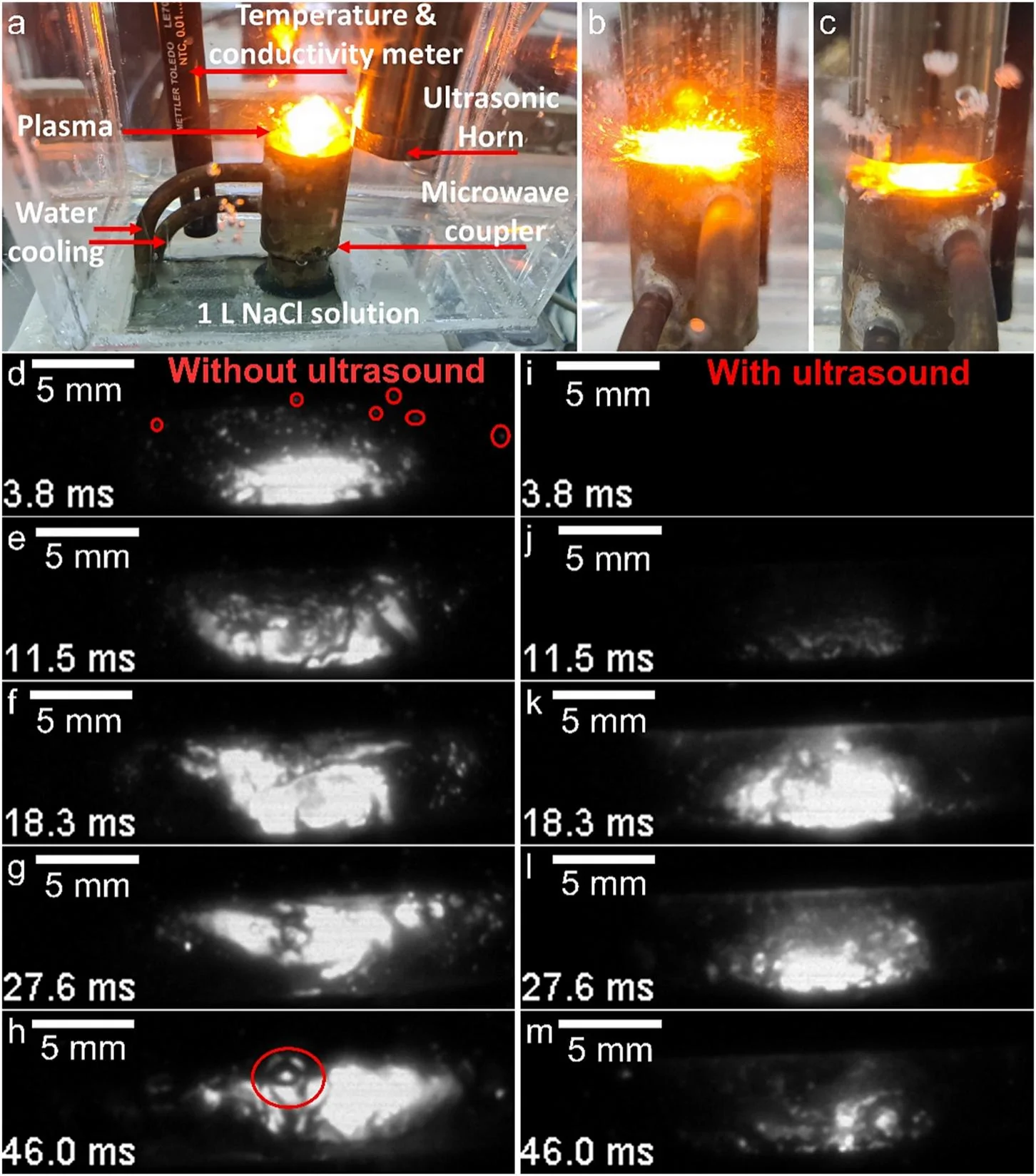
Comparison of microwave discharges with and without ultrasound. (a) Experimental setup and discharge (time-integrated image acquired over 10 ms) without sonotrode. Discharge with sonotrode at a distance of 5  mm from microwave coupler (b) without and (c) with ultrasound operation. Consecutive slow-motion images of the microwave discharge (d-h) without and (i-m) with ultrasound operation. Indicated are the times of the recordings during the application of a microwave burst of 50  ms. In (d), the red circles indicate small bubbles disconnected from the main vapor bubble containing the plasma discharge. In (h), the red circle indicates a lensing effect associated with deformation of the gas–liquid interface. Operating parameters: burst frequency $f_{r}=10\;Hz$, instantaneous microwave power $P_{i}=1950\;W$, duty ratio $d_{r}=50\;\%$, NaCl concentration [NaCl] = 10 g/L. The sonotrode was operated at 100 % amplitude.

The plasma appears bright yellow, likely due to emission from the sodium doublet around 589 nm, as observed in preliminary experiments and as reported by our colleague Timmermann et al. for a similar experimental setup, where optical emission spectroscopy using an AvaSpec-3648 spectrometer showed emission lines commonly observed in plasma–water interaction systems ([29], Fig. 5), including $H_{\alpha }$, $H_{\beta }$, OH radical emission lines, and the O emission line at 777 nm.

Fig. 2b shows the discharge with the sonotrode positioned 5 mm above the microwave coupler but without ultrasound. The plasma extends to the edge of the sonotrode, which is uniformly surrounded by a cloud of small bubbles, likely consisting of residual gas produced during the preceding discharges.

Fig. 2c was recorded during ultrasound operation, which confined the discharge to a smaller volume. Bubbles were observed rising and oscillating along the sonotrode and forming clusters. This clustering likely results from secondary Bjerknes forces [15], which tend to draw bubbles together in an acoustic field.

Slow-motion imaging allowed us to resolve the discharge dynamics with higher temporal resolution, both without ultrasound (Fig. 2, panels d-h) and with ultrasound (panels i-m). The corresponding slow-motion videos are provided in the supplementary material. Small bubbles (Fig. 2d, red circles) were observed near the larger vapor bubble that contained the plasma discharge and was directly connected to the top of the microwave coupler. For brevity, the larger vapor bubble is hereafter referred to as the “main bubble”. The small bubbles (Fig. 2d, red circles) appeared bright not because they contained plasma, but because their gas–liquid interfaces reflected the light emitted by the plasma discharge. A lensing effect can also be observed when the gas–liquid interface is suitably deformed, either due to deformation of the main bubble or to the presence of small bubbles located in front of the main bubble (Fig. 2h, red circle). Such lensing effects can complicate the identification of the main bubble shape and the quantification of its volume. They may also bias the quantification of the discharge light emission.

In addition, the slow-motion videos reveal that the discharges underwent short successive periods of extinction and re-illumination, which are not noticeably different with and without ultrasound. As already shown by the time-integrated recordings (Fig. 2, panels b-c), discharges were more confined when ultrasound was applied.

### 3.2 Synergy of plasma and ultrasound for H_2_O_2_ production

Fig. 3a shows the time-dependent accumulation of H_2_O_2_ in a 10 g/L NaCl solution for several treatment conditions. Ultrasound treatment at 100 % amplitude, in the absence of plasma, did not produce a detectable amount of H_2_O_2_ (as shown by the blue symbols and line in Fig. 3a). The H_2_O_2_ concentration increased with treatment time for all conditions involving plasma. The plasma-only treatment showed an approximately linear increase over the investigated treatment time. The combined plasma–ultrasound treatment (green symbols and line in Fig. 3a) showed a faster initial accumulation followed by a slower increase at longer treatment times, indicating a nonlinear temporal evolution. This behavior may result from a balance between H_2_O_2_ formation and consumption. As H_2_O_2_ accumulates, secondary reactions between H_2_O_2_ and plasma-generated hydroxyl and hydroperoxyl radicals may increasingly contribute to H_2_O_2_ consumption [2], [31]. This nonlinear behavior may also be influenced by the presence of recirculating flow, which could favor locally higher H_2_O_2_ concentrations and temperatures near the microwave discharge. Such conditions may enhance H_2_O_2_ decomposition or consumption.

**Fig. 3:**
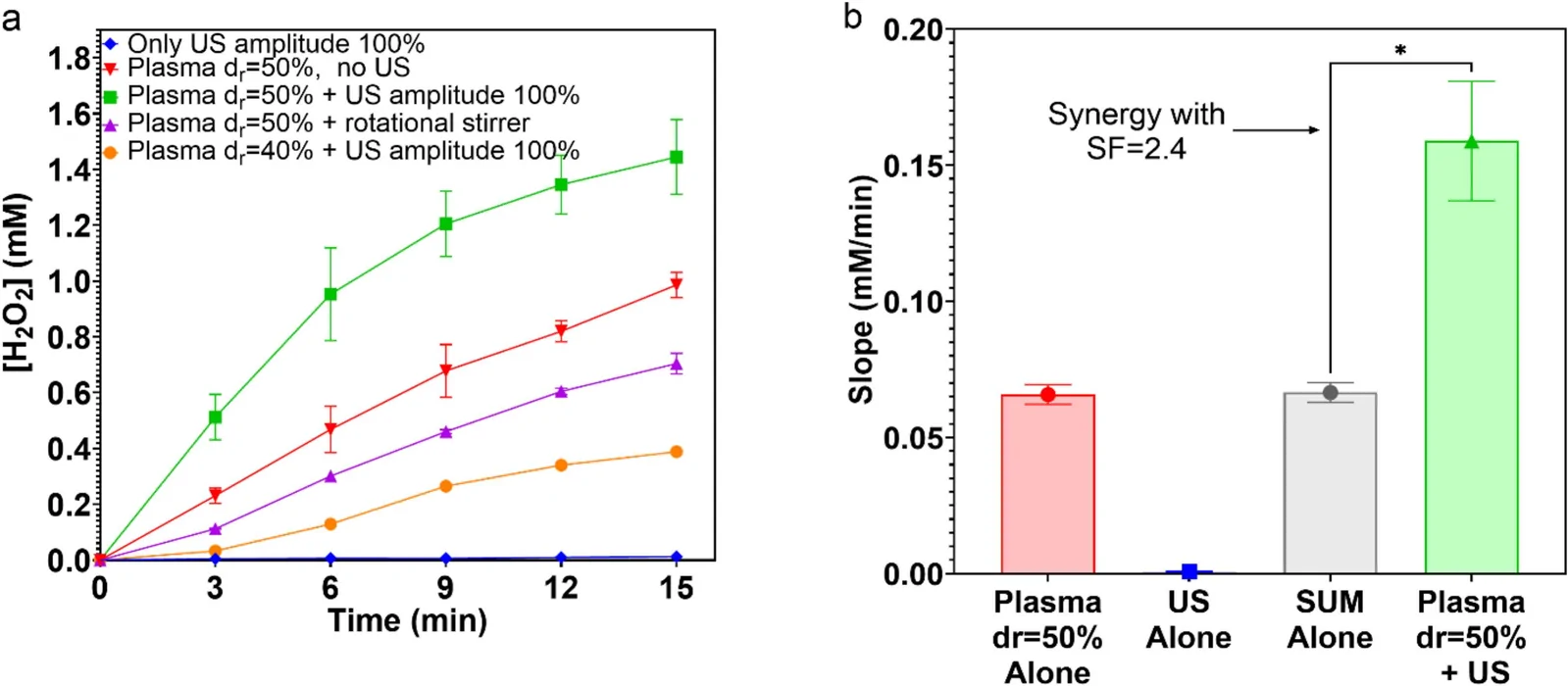
Synergistic enhancement of H_2_O_2_ production by ultrasound during underwater microwave plasma treatment. (a) Temporal evolution of H_2_O_2_ concentration for the five treatment conditions indicated in the legend. Microwave bursts were applied at a repetition frequency $f_{r}=10\;Hz$, a variable duty ratio $d_{r}$, and an instantaneous microwave power of $P_{i}=1950\;W$. The NaCl concentration was [NaCl]=10g/L. The sonotrode was positioned 5 mm above the microwave coupler and, when operated, was set to 100 % amplitude. Results represent averages and standard deviations of two independent experiments. (b) Initial H_2_O_2_ production rates obtained from linear fits of the early-time concentration profiles (≤6min for green graph,15 min otherwise). Bars represent the mean slopes, and error bars represent the standard deviations of the fitted slopes. SF indicates the synergy factor. The horizontal brackets indicate the statistical comparison using an unpaired *t*-test. Statistical significance is denoted as follows: ∗ p < 0.05, ∗∗ p < 0.01, and ∗∗∗ p < 0.001.

The treatment conditions can first be compared after 15 min of cumulative treatment. Ultrasound alone did not produce a detectable concentration of H_2_O_2_, whereas underwater microwave discharges generated a concentration of 0.9 mM under the following microwave burst parameters: $f_{r}=10\;\mathrm{Hz}$, $d_{r}=50\;\%$, $P_{i}=1950\;W$ (red symbols and line in Fig. 3a). When the same microwave discharge conditions were combined with sonication at 100 % amplitude, the H_2_O_2_ concentration reached 1.5 mM after 15 min and was higher than for all other conditions at each comparable sampling time (green symbols and line in Fig. 3a). This demonstrates that ultrasound synergistically enhanced H_2_O_2_ production during the plasma–liquid interaction.

The initial H_2_O_2_ production rates were also compared using linear fits of the concentration profiles. For the combined plasma–ultrasound treatment at $d_{r}=50\;\%$ (green symbols and line in Fig. 3a), the regression was restricted to the first 6 min because H_2_O_2_ accumulation became nonlinear at longer treatment times. For all other conditions, the concentration profiles remained approximately linear, and the regression was therefore performed over the full 15 min treatment period.

The slopes, corresponding to the apparent rates of H_2_O_2_ accumulation, were then extracted together with their standard deviations and are shown in Fig. 3b. From these slopes, a synergy factor (SF) was defined as: $\mathrm{SF}=s_{\mathrm{USP}}/\left(s_{\mathrm{US}}+s_{P}\right)$, where $s_{\mathrm{USP}}$ is the slope for the combined plasma–ultrasound treatment, $s_{\mathrm{US}}$ is the slope for ultrasound treatment alone, and $s_{P}$ is the slope for plasma treatment alone. A value of SF=1 would indicate the absence of synergy. Based on the results presented in Fig. 3b, SF=2.4, indicating that the combination of plasma and ultrasound led to an initial H_2_O_2_ production rate 2.4 times higher than the sum of the rates obtained with the individual treatments. Statistical testing showed that this difference was statistically significant with a p-value p=0.014.

Several hypotheses regarding the mechanisms underlying the synergy between plasma and ultrasound are explored in the Discussion section. However, one possible explanation is that the enhancement in H_2_O_2_ production results from increased macroscopic convection. To test this hypothesis, a basic control experiment was performed using mechanical stirring to generate a strong turbulent flow near the plasma-generated bubble. The H_2_O_2_ concentration was monitored while a rotational stirrer was operated at 300 rpm.

This particular experiment had a relatively modest objective: to test a limit condition by generating the strongest convection practically achievable in our setup. To this end, we selected the highest stirring rotation speed that did not cause liquid spillage, in order to maximize convection and favor turbulent flow conditions. Surprisingly, under these conditions, a significant decrease in H_2_O_2_ concentration was observed (purple symbols and line in Fig. 3). If a positive result had been obtained, i.e., if the H_2_O_2_ production rate in the presence of the stirred flow had been equal to or higher than that observed in the presence of ultrasound, we would have pursued systematic studies at different flow rates.

Moreover, the video available in the supplementary material shows that fluid motion and the flow induced by the stirrer altered the morphology of the discharge. Also, a large number of small bubbles were entrained by the rotational flow imposed by the stirrer and moved into close proximity of the microwave coupler. In the case of strong convection, the temperature gradients near the discharge change. This may result in a more pronounced cooling effect. As the temperature changes, so does the viscosity of the water, and consequently the formation of bubbles. This experiment does not exclude the possibility that other flow regimes, for example, generated using a nozzle immersed in water in front of the microwave coupler, and mimicking the flow structure induced by the sonicator, known as acoustic streaming [15], may reproduce the synergy observed in Fig. 3b.

To determine whether the microwave burst duty ratio was a critical parameter,$d_{r}$ was reduced from 50% to 40%. As a result, the average power delivered to the solution decreased by 20 %, since the burst duration was shortened, while the burst frequency remained constant. A significant reduction in H_2_O_2_ production was observed for $d_{r}=40\;\%$ (orange symbols and line in Fig. 3), accompanied by a noticeable change in discharge morphology: the discharge appeared less bright and occupied a smaller volume. This result is consistent with the expectation that lower energy input leads to reduced chemical activity. However, while the average microwave power decreased by only 20 %, the amount of H_2_O_2_ produced dropped by a factor of approximately seven compared to a duty ratio of $d_{r}=50\;\%$. To further investigate the role of burst duration, H_2_O_2_ production was also examined at constant average power but with varying burst frequency.

### 3.3 Operating parameters affecting H_2_O_2_ production

Several operating parameters can influence the synergistic enhancement of H_2_O_2_ production by plasma and ultrasound. We therefore investigated the effects of sonotrode position, ultrasound amplitude, microwave burst repetition frequency, and liquid conductivity. Unless otherwise specified, the microwave discharge was operated with 50 ms bursts, a duty ratio of $d_{r}=50\;\%$, and an instantaneous power of $P_{i}=1950$ W.

#### 3.3.1 Effect of sonotrode position

The highest rate of H_2_O_2_ production for the combination of plasma and ultrasound was achieved for the shortest investigated sonotrode–coupler distance of 5 mm. The H_2_O_2_ production rate decreased progressively with increasing sonotrode–coupler distance for distances of 10, 15, and 20  mm (Fig. 4). No substantial difference in H_2_O_2_ concentration was observed when the distance was further increased from 20  mm to 30  mm. The ultrasound was operated at 100 % amplitude for every distance. The local ultrasound amplitude at the plasma–liquid interface is expected to decrease with increasing distance, and acoustic streaming was affected concurrently [32]. Conversely, the volume that was available for plasma expansion was reduced for shorter distances. Still, the presence of ultrasound resulted in a slightly enhanced H_2_O_2_ production for treatment times shorter than 12 min, even for the largest distance.

**Fig. 4:**
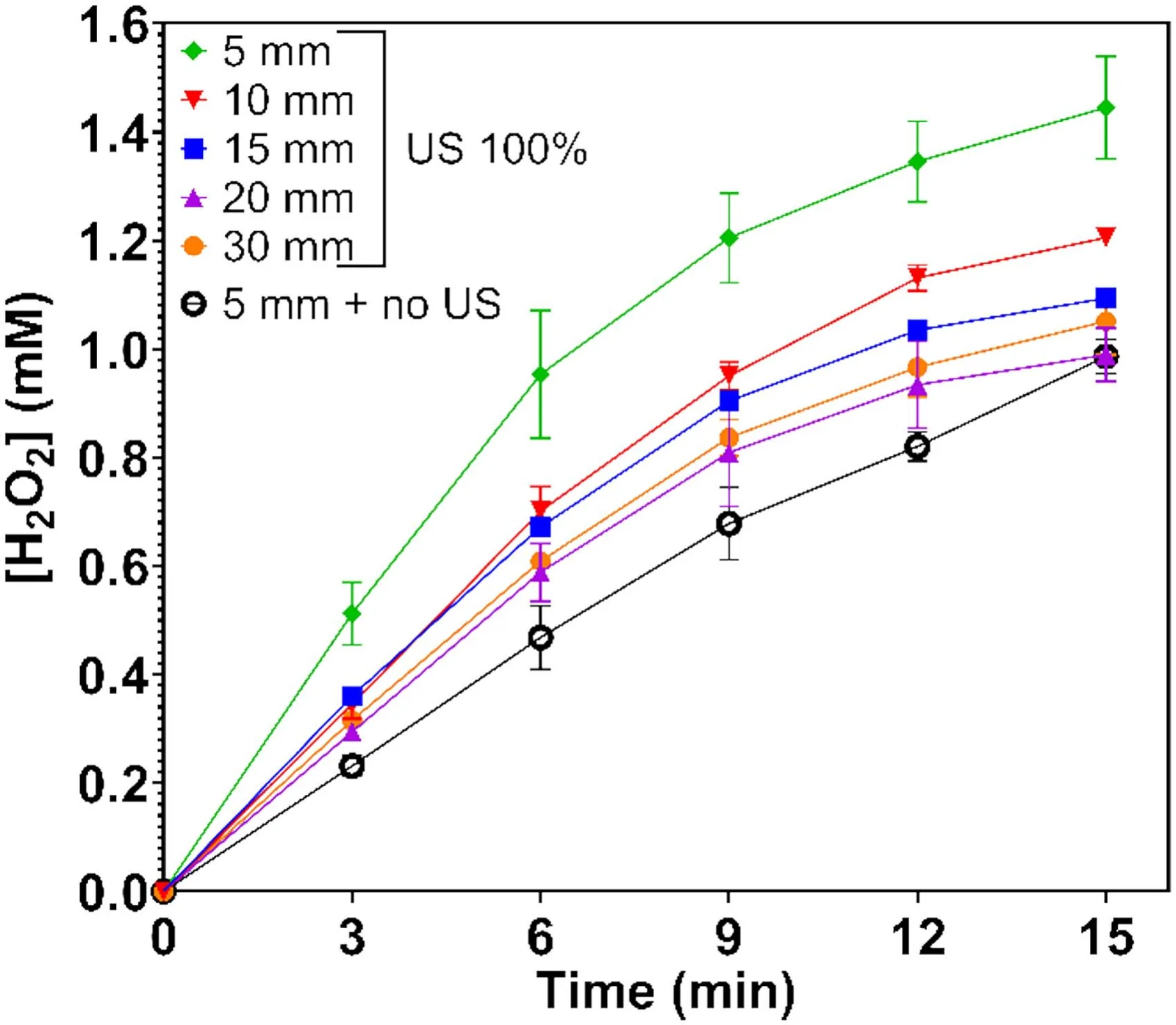
Temporal evolution of H_2_O_2_ concentration for different sonotrode distances from the top of the microwave coupler. The repetition rate and duty ratio for microwave bursts were 10 Hz and 50 %, respectively, for a NaCl concentration of [NaCl]=10g/L. The ultrasound amplitude was maintained at 100 %. Results represent averages and standard deviations of two independent experiments.

**Fig. 5:**
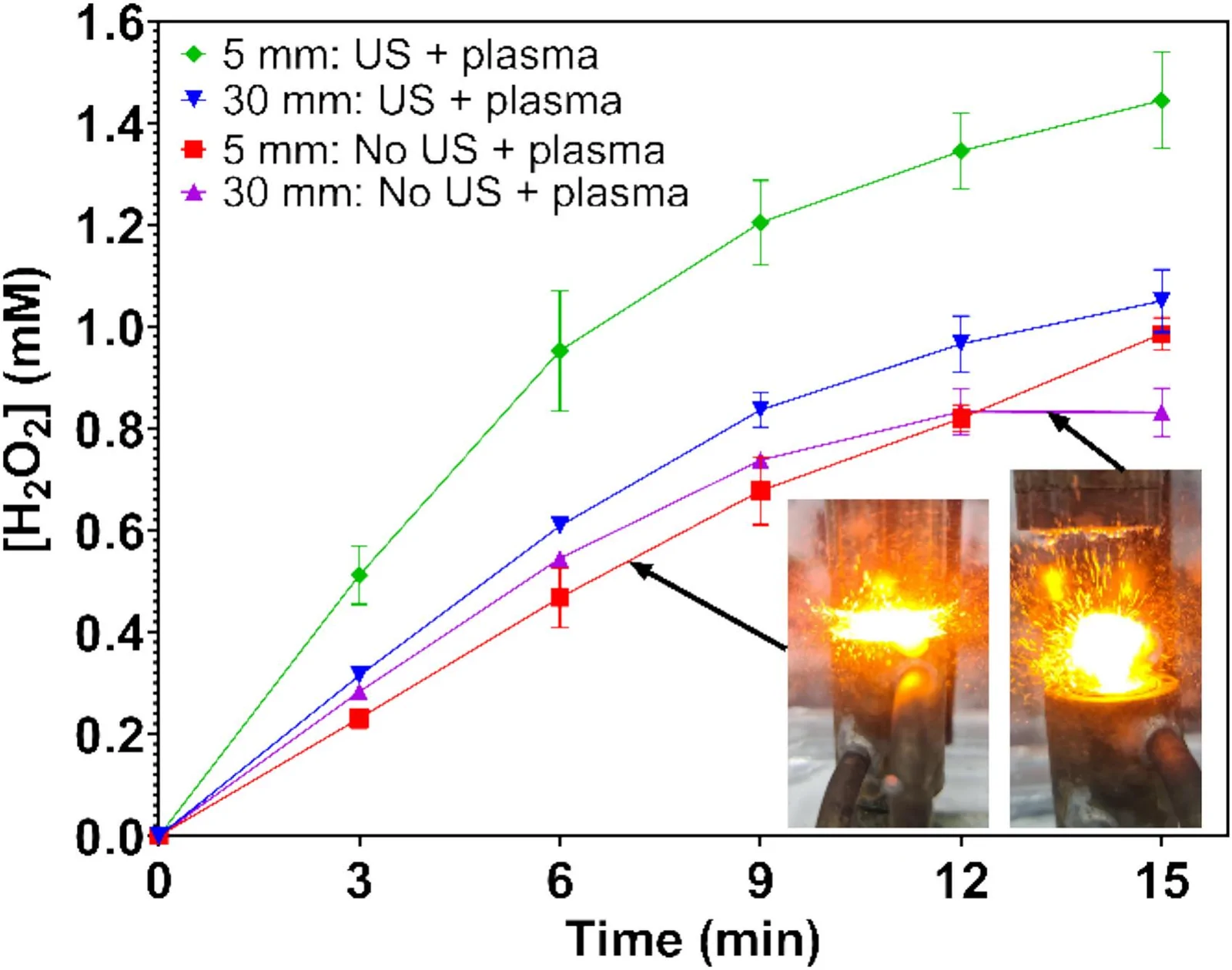
Temporal evolution of H_2_O_2_ concentration for two sonotrode positions (5 mm or 30 mm) with and without ultrasound operated at 100 % amplitude. Inset images correspond to the sonotrode positions 5 mm and 30 mm above the microwave coupler. The repetition rate and duty ratio for microwave bursts were 10 Hz and 50 %, respectively, for a NaCl concentration of [NaCl]=10g/L. Results represent averages and standard deviations of two independent experiments.

To determine whether the presence of the sonotrode itself, independent of position and of ultrasound operation, could influence H_2_O_2_ production, an experiment was conducted with the ultrasound turned off. Fig. 5 shows that, in the absence of ultrasound, H_2_O_2_ production is nearly identical, both at distances of 5  mm and 30  mm (red or purple symbols and lines in Fig. 5, respectively). This suggests that the mere presence of the sonotrode did not contribute to the enhanced H_2_O_2_ production that was observed during ultrasound operation (green or blue symbols and lines in Fig. 5, respectively).

Increasing the distance between the sonotrode and the discharge location had two main effects. First, the ultrasound intensity at the plasma was reduced, resulting in weaker microstreaming and smaller oscillations of the plasma–liquid interface. Second, it altered the pattern of acoustic streaming [15]. Similar changes also occurred when the ultrasound amplitude was reduced; therefore, a decrease in H_2_O_2_ production could be expected at lower intensity.

#### 3.3.2 Effect of ultrasound amplitude

Ultrasound amplitude plays a critical role in H_2_O_2_ production as shown in Fig. 6a. A decrease in ultrasound amplitude has a detrimental effect on H_2_O_2_ production. However, the H_2_O_2_ concentration does not decline steadily with decreasing intensity. Production remains relatively constant for amplitudes of 100 %, 75 %, and 50 %. A sharp drop in H_2_O_2_ concentration at 9 min is observed when the amplitude falls from 50 % to 25 %, although the concentration remains higher than in the absence of ultrasound. This threshold effect suggests that an amplitude of approximately 50 % is sufficient to induce a synergistic effect, and that the relationship between ultrasound amplitude and H_2_O_2_ production is nonlinear.

**Fig. 6:**
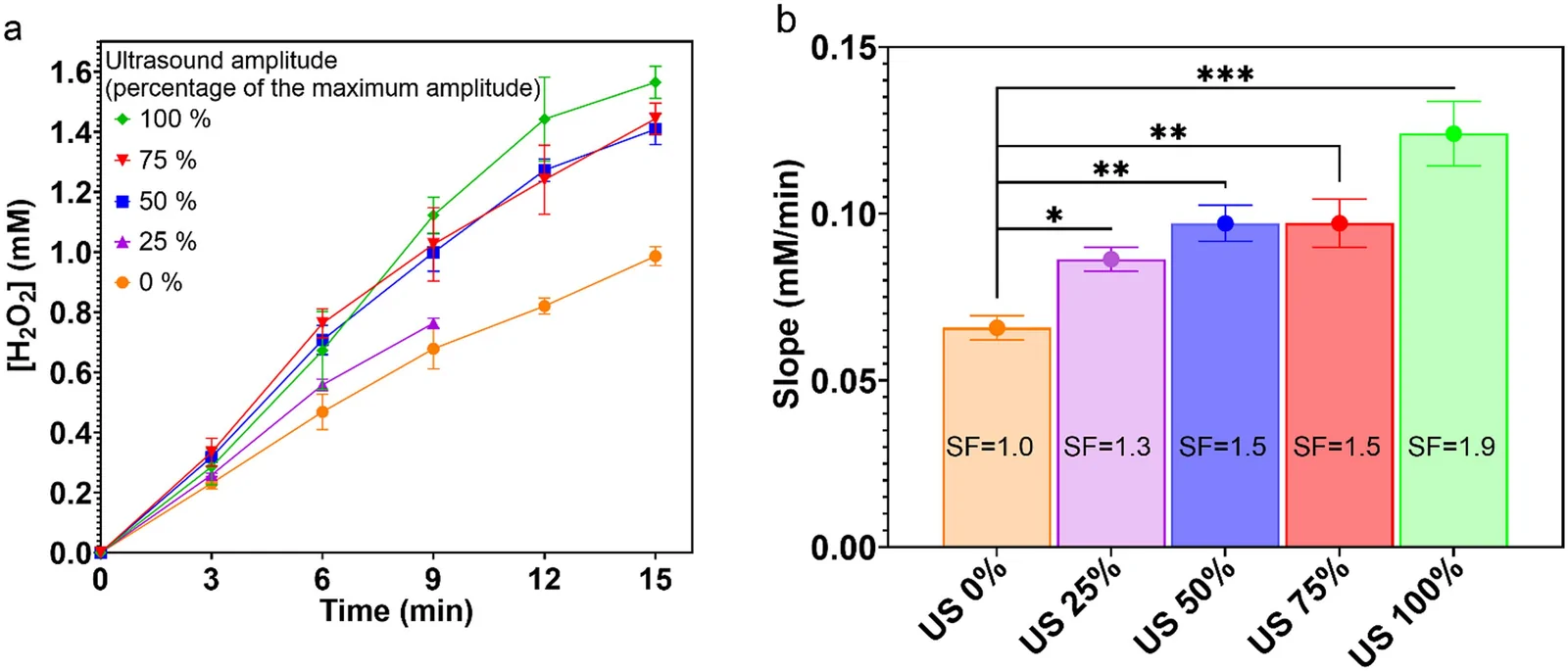
Effect of ultrasound amplitude on H_2_O_2_ production. (a) Temporal evolution of H_2_O_2_ concentration at different ultrasound amplitudes. The repetition rate and duty ratio for microwave bursts were 10 Hz and 50 %, respectively, for a NaCl concentration of [NaCl]=10g/L. The sonotrode was positioned 5 mm above the microwave coupler. Results represent averages and standard deviations of two independent experiments. (b) Initial H_2_O_2_ production rates determined from linear fits of the early-time concentration profiles (≤6 min for green graph; 15 min otherwise). Bars represent the mean slopes, and error bars represent the standard deviations of the fitted slopes. SF denotes the synergy factor. Horizontal brackets indicate pairwise statistical comparisons between groups. Statistical significance is denoted as follows: ∗ p < 0.05, ∗∗ p < 0.01, and ∗∗∗ p < 0.001. Comparisons were performed using an ordinary one-way ANOVA followed by Fisher’s uncorrected least significant difference (LSD) test.

Fig. 6b shows the initial H_2_O_2_ production rate, determined from the initial slope of the concentration–time curve, and the synergy factor (SF) defined in Section 3.2. The synergy remained statistically significant at an ultrasound amplitude of 25 % (p=0.02), while the initial production rate seems to vary smoothly with amplitude. The statistical significance of the synergy increased with ultrasound amplitude, consistent with the corresponding increase in the synergy factor.

#### 3.3.3 Effect of microwave burst repetition frequency

Microwave burst frequency is expected to affect H_2_O_2_ production. The number of bursts applied over a given time controls plasma formation and the duration of plasma–liquid interaction. A key question is whether a fixed energy input can be distributed over multiple bursts and how this influences H_2_O_2_ production. Therefore, the repetition frequency was varied from 10 to 40 Hz at a constant duty ratio ($d_{r}=50\;\%$), ensuring constant average power (Fig. 7). Accordingly, the burst duration was adjusted as a function of frequency to maintain a constant duty ratio, yielding 25 ms at 20 Hz, 16.7 ms at 30 Hz, and 12.5 ms at 40 Hz, compared to the standard condition of 50 ms bursts at 10 Hz.

**Fig. 7:**
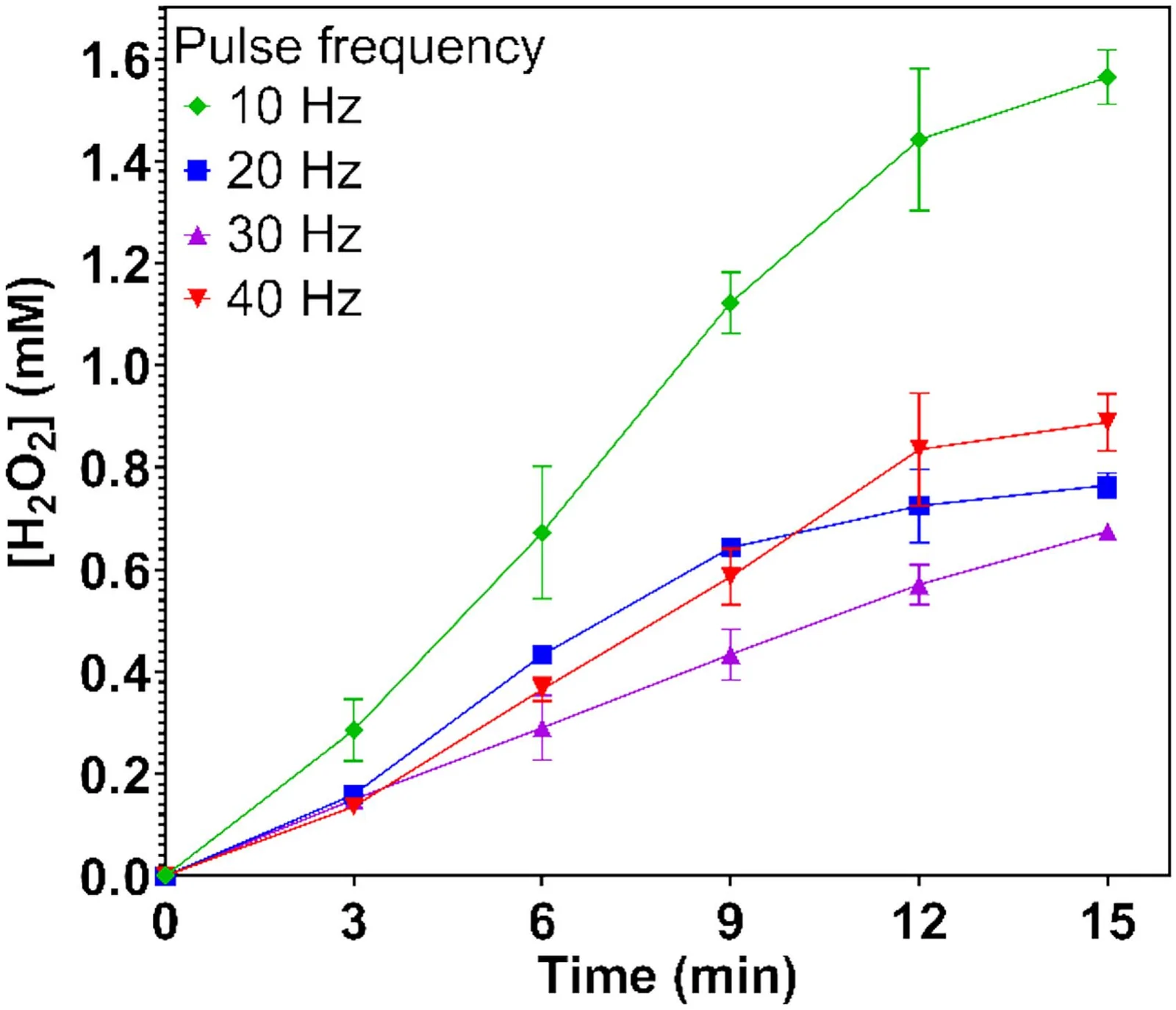
Temporal evolution of H_2_O_2_ concentration as a function of microwave burst repetition frequency at constant average microwave power. The duty ratio for microwave bursts was kept at 50 % for a NaCl concentration of [NaCl]=10g/L. The sonotrode was positioned 5 mm above the coupler and operated with an amplitude set to 100 %. Results represent averages and standard deviations of two independent experiments.

Shorter microwave bursts require repeated heating and vaporization of the liquid to sustain the plasma discharge. This likely increases the fraction of microwave energy consumed by heating and vaporization, thereby reducing the energy available for plasma formation within one microwave burst.

This might explain why H_2_O_2_ production significantly decreased at frequencies above 10 Hz. Interestingly, a repetition rate of 30 Hz was slightly less effective in generating H_2_O_2_ than 20 or 40 Hz. Thus, at constant average power, longer and less frequent microwave bursts favored H_2_O_2_ production. However, this has to be balanced against technical limitations, such as excessive heating of the liquid by the plasma and a possible erosion of plasma-facing components.

#### 3.3.4 Influence of NaCl concentration

Different NaCl concentrations were used to adjust the conductivity of the aqueous solution. A higher conductivity is a favorable condition for igniting and sustaining underwater microwave discharges. Additionally, the dynamics of the microwave discharge change depending on conductivity and liquid composition in general. With the current experimental setup and the maximum available power, plasma discharges could not be initiated in deionized water. At NaCl concentrations below 10 g/L, the discharge became unstable, with some microwave bursts failing to ignite the plasma and exhibiting stochastic on–off behavior across successive microwave bursts.

Conductivities, i.e., NaCl concentrations, determine the way that microwave energy is deposited in the solution. The investigated NaCl concentrations of 10, 15, 20, and 25 g/L corresponded to conductivities of 17.2, 24.3, 31.3, and 38.3 mS/cm at the reference temperature of 25 °C. Changes in electrical conductivity, or more broadly in the complex permittivity of the medium, should, therefore, to some extent alter microwave absorption, in particular the microwave absorption length. Due to heating, the electrical conductivity necessarily changed over the course of long treatment times. For example, at 40 °C, the conductivity was approximately 30 % higher than at 25 °C. Note that the tuning components of the microwave circuit were adjusted to maximize microwave absorption for each initial salt concentration, with tuning performed over a timescale of approximately 1–2 min.

Interestingly, increasing the NaCl concentration, and thus the liquid conductivity, was associated with a decrease in H_2_O_2_ production despite the expected increase in microwave energy deposition (Fig. 8). This indicates that the effect of dissolved ions cannot be explained by conductivity alone and likely involves additional chemical or plasma-related processes. The dissolved ions might have scavenged H_2_O_2_ precursors either in the plasma or subsequently in the bulk liquid. According to this hypothesis, H_2_O_2_ production would be expected to decrease continuously with increasing NaCl concentration. However, at concentrations above 20 g/L, H_2_O_2_ production stabilized and did not decline further. An alternative interpretation would be that the higher energy absorbed by the liquid and/or plasma due to higher conductivities may favor reaction pathways that do not lead to H_2_O_2_ formation [33]. Further experiments involving the adjustment of electrical conductivity with other salts could provide better insights into the underlying mechanisms.

**Fig. 8:**
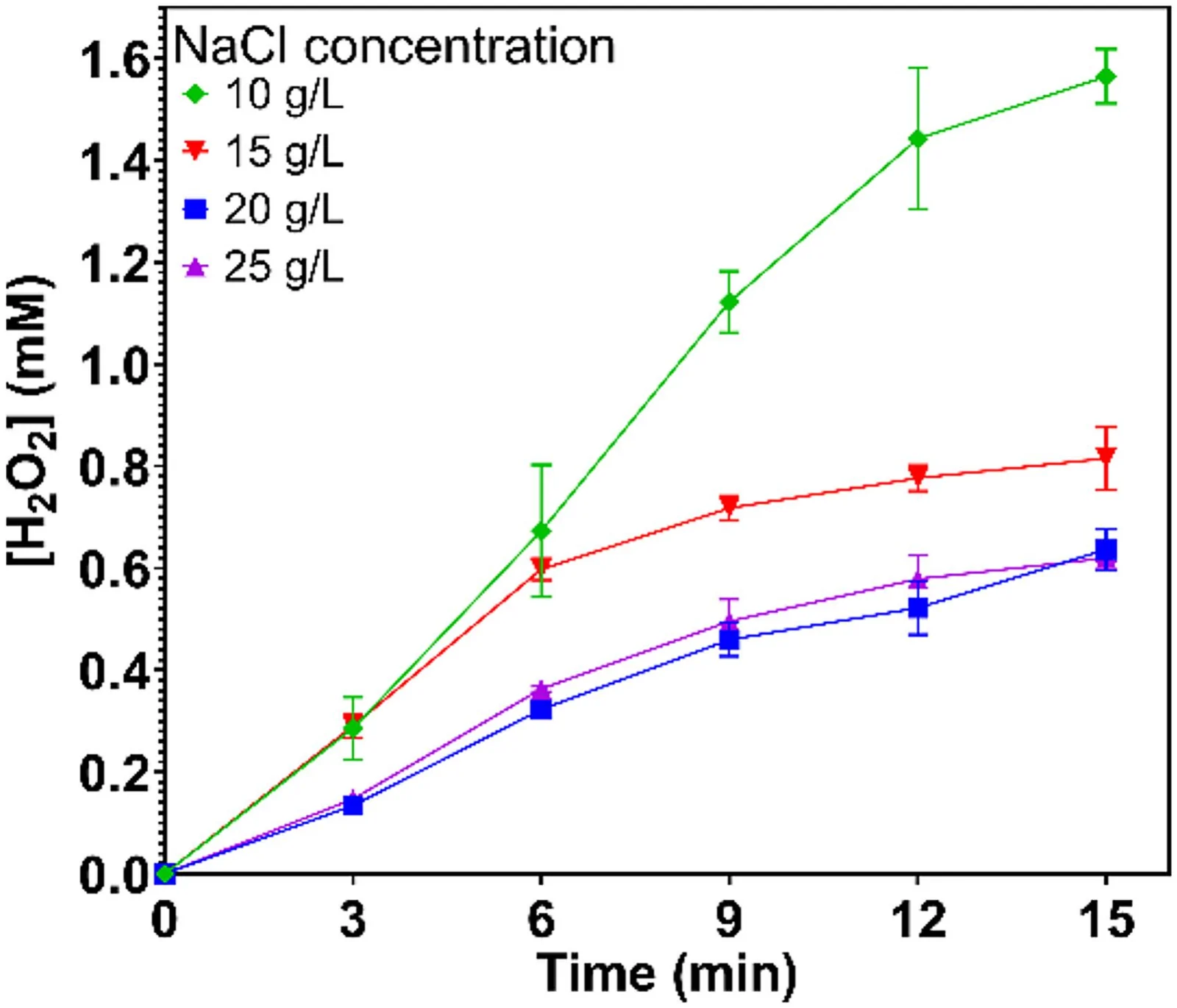
Temporal evolution of H_2_O_2_ concentration as a function of NaCl concentration. The repetition rate and duty ratio for microwave bursts were 10 Hz and 50 %, respectively. The sonotrode was positioned 5 mm above the coupler and operated with an amplitude set to 100 %. Results represent averages and standard deviations of two independent experiments.

### 3.4 Correlation between microwave energy, optical emission, and H_2_O_2_ production

The liquid medium absorbs microwaves differently depending on whether it is in a liquid, gaseous, or plasma state. Moreover, when a plasma develops, its density and the shape of the discharge affect the absorption behavior. Accordingly, microwave absorption is neither complete nor constant over time during a microwave burst (Fig. 9). The reflected power was used as an indicator of microwave coupling under the different operating conditions. However, interpretation of reflected power is challenging because the reflective properties of the coupler depend on the dielectric characteristics of the medium above the ceramic. Reflected power is also affected by settings of the tuning component, which determine how efficiently the incident energy is coupled into the system. Although reflected power cannot be interpreted unambiguously, the reflected power serves as a parameter for controlling repeatability and optimizing energy absorption.

**Fig. 9:**
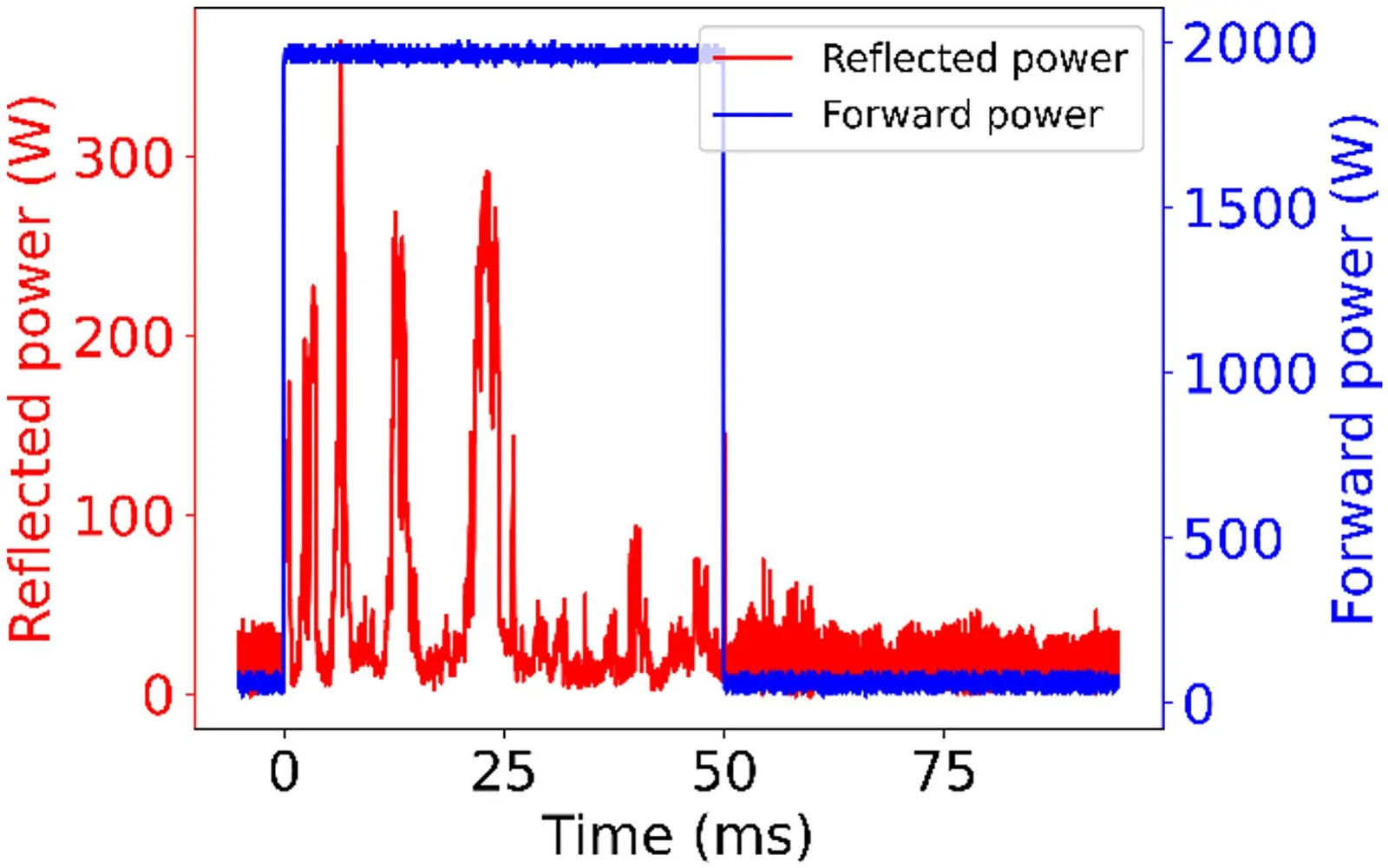
Instantaneous forward and reflected microwave power during one microwave burst of 50  ms. The repetition rate and duty ratio for microwave bursts were 10 Hz and 50 %, respectively, for a NaCl concentration of [NaCl]=10g/L. The sonotrode was positioned 5 mm above the coupler and operated with an amplitude set to 100 %.

During specific experiments, slow-motion imaging and oscilloscope measurements were performed to investigate potential correlations between H_2_O_2_ generation, reflected microwave energy, and discharge morphology.

Table 1 summarizes the reflected microwave energy per microwave burst, and the time-integrated light emission (expressed in arbitrary units, *a.u.*) for various treatment conditions. Values that have been changed for particular experiments in comparison to standard operating parameters are underlined. The light emission served as a rough indicator for the intensity and general evolution of the microwave discharge. The reflected energy was always less than 7 % of the incident microwave energy per burst. Under optimal conditions for H_2_O_2_ production (Table 1, ID II), the reflected energy per burst was minimal, with only 2.4J of the 98 J incident energy being reflected.

**Table 1:** Reflected energy per microwave burst and time-integrated light emission from the discharge under various experimental conditions (ID I to ID VIII). Mean values and standard errors for the energy were calculated from 200 bursts and for the time-integrated light emission from 20 measurements. Experimental conditions are defined by NaCl concentration, microwave burst frequency ($f_{r}$), duty ratio ($d_{r}$), ultrasound amplitude, and sonotrode position. Values that have been changed for specific experiments in comparison to standard operating parameters are underlined.

ID	[NaCl] g/L	$f_{r}$Hz	$d_{r}$%	Ultrasound Amplitude %	Sonotrode position mm	Reflected energy per burst (J)	Forward energy per burst (J)	Reflected power %	time-integrated light emission (a.u)	Average H_2_O_2_ concentration (mM) after 15 min
I	10	10	50	**0**	5	3.9±0.3	98	4.0	2.9±0.5	0.99
II	10	10	50	100	5	2.4±0.2	98	2.4	1.4±0.4	1.45
III	10	10	50	0	**without sonotrode**	5.1±0.4	98	5.2	2.6±0.5	0.83
IV	10	10	50	**25**	5	6.6±0.5	98	6.7	1.0±0.2	−
V	10	10	50	**50**	5	3.2±0.2	98	3.3	1.9±0.2	1.41
VI	**15**	10	50	100	5	3.5±0.2	98	3.5	0.59±0.1	0.82
VII	10	10	**40**	100	5	7.0±0.5	78	9.0	0.42±0.06	0.39
VIII	10	**20**	50	100	5	2.8±0.2	49	5.7	0.18±0.03	0.77

#### 3.4.1 Impact of ultrasound operation

The comparison of experiments (ID I and ID II in Table 1) reveals that ultrasound operation reduced the reflected energy from 3.9 J to 2.4 J per burst, indicating improved microwave coupling. This reduction in reflected energy coincided with a strong enhancement in H_2_O_2_ production (c.f. Fig. 3), which supports the observed synergy between plasma and ultrasound. Correspondingly, the integrated light emission decreased from 2.9 ± 0.5 *a.u.* to 1.4 ± 0.4 *a.u.* The lower emissions suggest that the plasma may have been less stable or more confined within a smaller bubble when ultrasound was applied. This observation is consistent with Fig. 2, which shows that, in the absence of ultrasound, the plasma formed in larger bubbles and ignited sooner (within a microwave burst).

#### 3.4.2 Impact of sonotrode position

Removing the sonotrode (ID III in Table 1) resulted in an increase in reflected energy from 3.9 J to 5.1 J per microwave burst. This suggests that the sonotrode, being a metallic structure and a potential microwave reflector, interacts with the microwave circuit and alters the energy coupling. However, no significant changes were observed in H_2_O_2_ production or light emission (2.6 ± 0.5 *a.u.* compared to 2.9 ± 0.5 *a.u.*), indicating that the passive presence of the sonotrode itself, without ultrasound, has a minimal effect on the plasma reactivity and light emission of the discharge. This control demonstrates that the observed synergistic effect has to be directly attributed to the presence of ultrasound.

#### 3.4.3 Impact of ultrasound amplitude

Increasing the ultrasound amplitude from 25 % (ID IV in Table 1) to 100 % (ID II in Table 1) led to a notable decrease in reflected energy (6.6 J to 2.4 J per burst) and an increase in H_2_O_2_ production (comparison of experiments ID I, II, IV, V in Table 1 and Fig. 6). Concurrently, the integrated light emission remained lower than without ultrasound, with values of 1.0±0.2a.u*.* at 25 % amplitude and 1.4±0.4a.u*.* at 100 % amplitude. Interestingly, H_2_O_2_ production and reflected energy were comparable at 50 % and 100 % ultrasound amplitudes (ID V vs ID II in Table 1), which may suggest a threshold effect with no further improvement for higher ultrasound amplitude (as further explained in the Discussion section).

#### 3.4.4 Impact of microwave burst repetition frequency

Increasing the microwave burst repetition frequency from 10 Hz (ID II in Table 1) to 20 Hz (ID VIII in Table 1) halved the microwave burst duration and, accordingly, the incident microwave energy per burst (49 J). There was a slight increase in reflected energy per burst (from 2.4±0.2J to 2.8±0.2J), but a marked decrease in the emitted light (1.4 ± 0.4 *a.u.* to 0.18 ± 0.03 *a.u.*). The shorter burst duration of 20 Hz reduced H_2_O_2_ production and light emission considerably, even though the average delivered microwave power was the same. As previously described, the power diverted to liquid heating and evaporation also reduces the energy available for plasma heating.

#### 3.4.5 Impact of microwave burst duty ratio

Reducing the duty ratio from 50 % (ID II in Table 1) to 40 % (ID VII in Table 1) for a microwave burst repetition frequency of 10 Hz caused a substantial increase in reflected energy (2.4  J compared to 7.0 J per burst) and a sharp decline in H_2_O_2_ production. This was accompanied by a significant reduction in light emission (1.4 ± 0.4 *a.u.* to 0.42 ± 0.06 *a.u.*), indicating that the plasma is less stable and emits less light when less energy is available for sustaining the discharge.

#### 3.4.6 Impact of NaCl concentration

An increase in NaCl concentration from 10 g/L (ID II in Table 1) to 15 g/L (ID VI in Table 1) resulted in a slight increase in reflected energy (2.4 J to 3.5 J per burst) and a significant decrease in H_2_O_2_ production. As described above, this effect cannot necessarily be attributed solely to the deposited energy; changes in plasma-chemical reaction pathways induced by the presence of salt must also be considered. The integrated light emission decreased drastically (1.4 ± 0.4 *a.u.* to 0.59 ± 0.1 *a.u.*), suggesting that the plasma chemistry may also have been affected.

#### 3.4.7 Insights from light emission and reflected microwave power

In summary, reflected power and time-integrated light emission do not generally correlate with H_2_O_2_ production. However, a substantial decrease in H_2_O_2_ production was associated with a significant drop in light emission (< 1.0 *a.u.*).

For otherwise comparable operating conditions during ultrasound operation, a lower reflected power was associated with an increase in H_2_O_2_ production. Therefore, this parameter can be utilized to optimize H_2_O_2_ production to some extent.

The supplementary material includes slow-motion sequences illustrating the different experimental conditions described in Table 1. Without ultrasound, a large gas bubble was formed, with the plasma discharge occupying only a portion of its volume. In contrast, when ultrasound is applied, large bubbles either do not form or are rapidly broken apart.

Fig. 10 shows the temporal evolution of the cumulative light emission. Each curve corresponds to one of 20 individually recorded discharges. These discharges were sampled randomly throughout the whole treatment time and do not represent consecutive events.

**Fig. 10:**
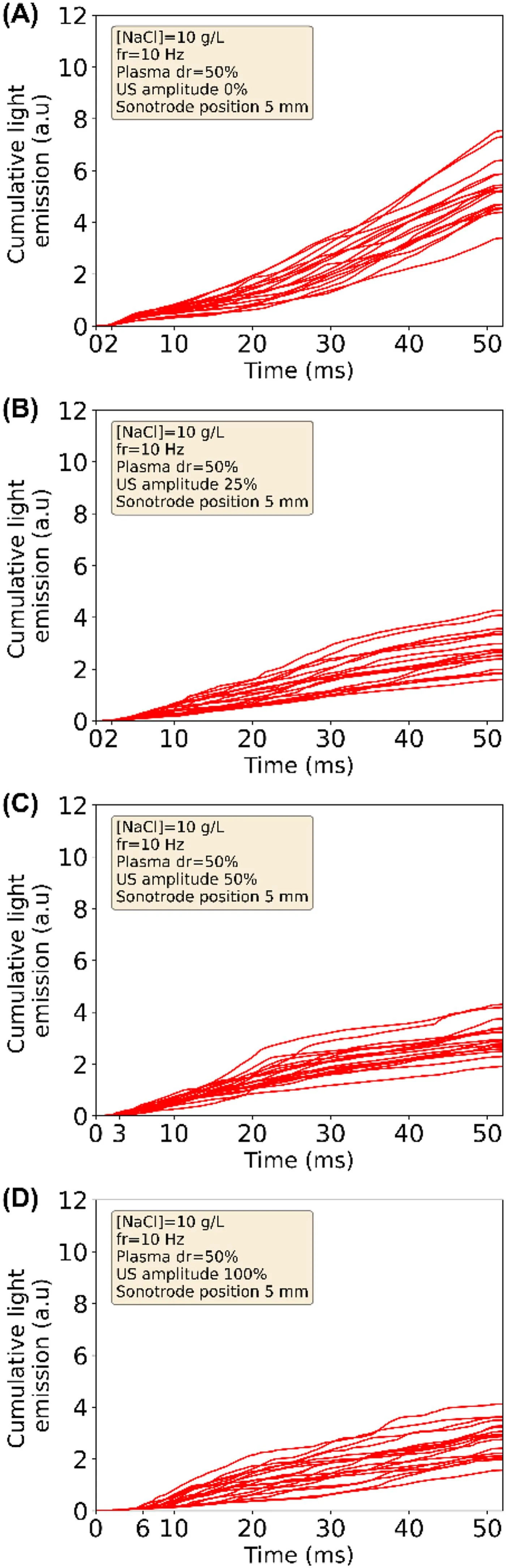
Cumulative light emission over time (a.u.) from discharges operated in combination with different ultrasound amplitudes: (A) 0 %, (B) 25 %, (C) 50 %, and (D) 100 % in a NaCl solution with [NaCl]=10g/L. The sonotrode was positioned 5 mm above the coupler. The repetition rate and duty ratio for microwave bursts were 10 Hz and 50 %, respectively. Each red curve represents an individual discharge.

The ignition delay $\tau_{\mathrm{igni}}$ is defined as the time interval between the onset of the microwave burst and the first detectable light emission. Without ultrasound, the ignition delay $\tau_{\mathrm{igni}}$ is 2 ms (Fig. 10A). When ultrasound is applied at 100 % amplitude, the ignition delay $\tau_{\mathrm{igni}}$ increases to 6 ms (Fig. 10D). In contrast, ultrasound at 25 % amplitude (Fig. 10B) results in only a slight increase in ignition delay beyond 2 ms, while a 50 % amplitude (Fig. 10C) yields an ignition delay of approximately 3 ms.

## 4 Discussion

Under the treatment conditions that led to the highest H_2_O_2_ production rate (ultrasound amplitude of 100 % at 5 mm distance, repetition frequency of 10 Hz, $d_{r}=50\;\%$, and $\left[NaCl\right]=10\;g/L$), an average H_2_O_2_ production rate of $0.20\;g.h^{-1}$ over the 15-min treatment was obtained. Without ultrasound, the production rate was only $0.13\;g.h^{-1}$. This corresponds to a +47% enhancement attributable to ultrasound.

When accounting for both microwave power absorption and the ultrasound power consumption, the H_2_O_2_ yields are $0.18\;g.{\mathrm{kWh}}^{-1}$ and $0.14\;g.{\mathrm{kWh}}^{-1}$ with and without ultrasound, respectively. This represents a 26 % enhancement in yield attributable to ultrasound (Table 2). An ultrasound amplitude of 50 % still led to a significant increase in H_2_O_2_ production with a lower ultrasonic power transmitted to the solution (49.3 W). This resulted in a slightly higher H_2_O_2_ yield, which was 7 % higher than that obtained at 100 % amplitude.

**Table 2:** Average H_2_O_2_ production rate and energy yield calculated over 15 min of treatment as a function of the applied ultrasound amplitude and plasma operation. Microwave bursts were applied at a repetition frequency of 10 Hz and a duty ratio of 50 %. Experiments were performed at a NaCl concentration of 10 g/L, with the sonotrode positioned 5 mm above the microwave coupler. Values are reported as averages of two independent experiments. Values reported as 0 indicate that H_2_O_2_ production was below the limit of detection.

Ultrasound amplitude %	Plasma	H_2_O_2_ production rate g/h	H_2_O_2_ Yield$g.kWh^{-1}$
100 %	ON	0.20	0.18
50 %	ON	0.19	0.19
0 %	ON	0.13	0.14
100 %	OFF	0	0

These results clearly indicate enhanced H_2_O_2_ production and, more importantly, suggest a synergistic effect between plasma and ultrasound, as no detectable H_2_O_2_ production was observed with ultrasound treatment alone. However, the underlying mechanisms responsible for the observed synergy cannot be fully elucidated based on the limited set of experiments and parameters investigated. A comprehensive understanding would require a broader range of experimental conditions, including different ultrasound frequencies and thus alternative sonotrodes and sonicators, as well as comparisons with other plasma sources. Such an extensive study is beyond the scope of the present work, which was originally aimed at evaluating improvements in microwave-generated plasma in liquids for practical applications.

Several mechanisms may account for the synergy in H_2_O_2_ production.

### 4.1 Transport from the plasma–liquid interface to the bulk liquid

Ultrasound may enhance the transport of reactive chemical species from the plasma–liquid interface to the bulk of the liquid. High-amplitude ultrasound can induce microstreaming, which enhances mass transfer in the liquid [34]. According to this hypothesis, microstreaming could have facilitated the extraction of H_2_O_2_ and other long-lived species from the plasma–liquid interface into the liquid by reducing the boundary layer thickness [34].

### 4.2 Transport from the plasma core to the plasma–liquid interface

Ultrasound may also enhance the transfer of H_2_O_2_ formed in the gaseous state at the core of the plasma to the plasma–liquid interface. The plasma–liquid interface oscillated at a frequency of 20 kHz (superimposed by the ultrasound), and this movement may have increased the kinetic of solubilization of H_2_O_2_ at the interface.

### 4.3 Macroscopic convection

The operation of the sonotrode generates a macroscopic flow, repelling liquid from its surface and creating a steady flow known as acoustic streaming [15]. Given that the microwave coupler is positioned beneath the sonotrode, the resulting convection could contribute to the observed synergy by improving H_2_O_2_ transport from the plasma–liquid interface relative to diffusion alone. An experiment designed to reproduce flow patterns characteristic of ultrasonic streaming could provide further insight. Precise measurement and controlled replication of the flow field generated by the sonotrode would allow this hypothesis to be rigorously tested.

### 4.4 Plasma chemistry

Although ultrasound does not propagate through the plasma due to the significant mismatch in acoustic impedance between the plasma and liquid, oscillations of the plasma–liquid interface will induce pressure variations within the plasma bubble. These fluctuations, synchronized with the phase of the ultrasonic wave, may have promoted specific chemical pathways that could enhance H_2_O_2_ production during certain phases of the incident ultrasound wave. Because plasma-chemical reaction rates and pathways are pressure dependent, these oscillations could modify the chemistry within the plasma bubble.

Time-resolved plasma diagnostics could provide further insight into whether key parameters, such as electron density and excited species populations, vary significantly on the timescale of the ultrasound period.

### 4.5 Nebulization at the plasma–liquid interface

Since the large bubble generated by the microwave discharge formed close to the sonotrode, and since the ultrasound amplitude was high enough to cause cavitation, it is likely that some water was nebulized from the liquid interface within the plasma bubble. Water droplet nebulization is a well-known mechanism that enhances mass transfer of reactive species such as H_2_O_2_ from the plasma to the liquid phase [35].

According to Colmenares and Chatel [17], the average diameter D of droplets formed during nebulization depends on the surface tension γ and density ρ of the liquid, as well as the ultrasound frequency f, and can be estimated using the Lang equation [36]:

(1) $$D=0.34{\left(\frac{8\pi \gamma }{\rho f^{2}}\right)}^{1/3}$$

For an ultrasound frequency of 20 kHz, in water the resulting droplet diameter would be approximately D=58μm. This is significantly smaller than the plasma-containing bubbles observed, which are typically in the millimeter to centimeter range, and also not detectable with the present experimental setup. High-resolution imaging would be required to assess whether nebulization occurs within the plasma bubble.

To the best of our knowledge, neither the results of the present study nor the available literature, as summarized for example in the review by Locke and Shih [2], allow one mechanism to be favored over another. Among the 78 H_2_O_2_ production rates reported for different plasma sources in [2], only eight are equal to or higher than those reported here. However, the energy yield obtained in the present study is lower than the lowest yield reported in these studies.

Considering the diversity of experimental systems and approaches, this likely reflects differences in plasma exposure conditions and underlying processes. A more systematic comparison is therefore needed, especially to evaluate potential synergies between plasma and ultrasound for H_2_O_2_ production. It remains an open question whether such synergies occur for other plasma sources.

For microwave underwater discharges, the dominant pathway for H_2_O_2_ formation remains unclear. It is not yet established whether H_2_O_2_ is primarily formed in the gas phase and subsequently transferred to the liquid, as proposed by Winter et al. [5], or generated directly in the liquid phase through secondary reactions involving plasma-produced species such as solvated electrons ($e_{\mathrm{aq}}^{-}$) or hydroxyl radicals, as suggested by Locke and Shih [2].

Future studies should focus on the more fundamental aspects of plasma–ultrasound interactions. The presented system, which produces a transient plasma that expands within the liquid, is not ideally suited for investigations at the plasma–liquid interface. A more stable plasma–liquid interface and a continuous plasma source would enable clearer insights into the underlying mechanisms. A more detailed understanding of plasma chemistry would also require investigating the role of other reactive species, such as nitrates, nitrites, and OH radicals. It remains unclear whether these species are similarly affected by combined plasma–ultrasound treatment. Future studies should also investigate the effect of different salts used to adjust conductivity on plasma chemistry. These insights could ultimately help optimize plasma–liquid interface chemistry for the intended application and improve the yield of plasma–liquid systems, while also clarifying whether ultrasound modifies plasma chemistry beyond its effect on H_2_O_2_ formation.

## 5 Conclusion

The study demonstrates the synergistic interaction between ultrasound and underwater microwave plasma in enhancing H_2_O_2_ production in NaCl solutions. Systematic variations in ultrasound amplitude, sonotrode position, and salt concentration allowed us to determine how they affect both the efficiency of H_2_O_2_ generation and the discharge behavior. Overall, the main results of this study show the potential of combining ultrasound with underwater plasma discharges to enhance H_2_O_2_ production rate and yield. This offers promising opportunities for advancing plasma-based applications in water decontamination, chemical synthesis, and also the efficient generation of reactive species for plasma medicine. Accordingly, the combination of ultrasound and plasma has the potential to become a standard approach for enhancing advanced oxidation processes. The preliminary results presented here clearly indicate that achieving higher yields and a deeper understanding of the mechanisms underlying plasma–ultrasound synergy will require further research.

## CRediT authorship contribution statement

**B. Honnorat:** Writing – review & editing, Writing – original draft, Visualization, Validation, Methodology, Investigation, Data curation, Conceptualization. **V. Brüser:** Writing – review & editing, Supervision, Resources, Project administration, Conceptualization. **J.F. Kolb:** Writing – review & editing, Supervision, Resources, Project administration, Conceptualization.

## Funding

This research has been supported by the Leibniz Collaborative Excellence Programme, Application number: K494/2022.

## Declaration of competing interest

The authors declare that they have no known competing financial interests or personal relationships that could have appeared to influence the work reported in this paper.

## Data Availability

Mendeley Datasupplementary material Ultrasound-Enhanced Hydrogen Peroxide Generation of Microwave Plasma Discharges in Conductive Aqueous Solutions (Original data)
